# Near atmospheric carbon dioxide activates plant ubiquitin cross-linking

**DOI:** 10.1016/j.bbadva.2023.100096

**Published:** 2023-06-17

**Authors:** Harry G Gannon, Martin J Cann

**Affiliations:** a Department of Biosciences, Durham University, South Road, Durham DH1 3LE, UK; b Biophysical Sciences Institute, Durham University, South Road, Durham DH1 3LE, UK

**Keywords:** Carbon dioxide, Carbamate, Ubiquitin, Arabidopsis

## Abstract

Background Identifying CO_2_-binding proteins is vital for our knowledge of CO_2_-regulated molecular processes. The carbamate post-translational modification is a reversible CO_2_-mediated adduct that can form on neutral *N*-terminal α-amino or lysine ε-amino groups.

Methods We have developed triethyloxonium ion (TEO) as a chemical proteomics tool to trap the carbamate post-translational modification on protein covalently. We use ^13^C-NMR and TEO and identify ubiquitin as a plant CO_2_-binding protein.

Results We observe the carbamate post-translational modification on the *Arabidopsis thaliana* ubiquitin ε-amino groups of lysines 6, 33, and 48. We show that biologically relevant near atmospheric PCO_2_ levels increase ubiquitin conjugation dependent on lysine 6. We further demonstrate that CO_2_ increases the ubiquitin E2 ligase (*At*UBC5) charging step via the transthioesterification reaction in which Ub is transferred from the E1 ligase active site to the E2 active site.

Conclusions and general significance Therefore, plant ubiquitin is a CO_2_-binding protein, and the carbamate post-translational modification represents a potential mechanism through which plant cells can respond to fluctuating PCO_2_.

## Highlights

• Plant ubiquitin binds carbon dioxide.
• Carbon dioxide upregulates ubiquitin conjugation dependent on lysine 6.
• Carbon dioxide activates E2 ligase charging with ubiquitin.

## 1 Introduction

CO_2_ was first discovered in 1757 as a component of the gas exhaled from the lung [1]. It is now broadly recognised as a vital component of various physiological processes. It has roles in, for example, metabolism, photosynthesis, chemosensing, and cellular homeostasis [2]. The fundamental importance of the gas to biology has meant that organisms across kingdoms can sense and adapt to fluctuating CO_2_ [3]. Unfortunately, our knowledge of direct CO_2_ targets is scant compared to our extensive knowledge of its impact on physiology. Knowledge of direct CO_2_ targets in photosynthetic organisms is essential for exploiting them for green biotechnology and addressing crop responses to climate change.

We have a relatively large knowledge base for CO_2_ roles in cell, tissue and organismal physiology, e.g., metabolism, acid-base homeostasis, and transport [2,4]. We have a smaller knowledge base of CO_2_-responsive signalling pathways. These include, for example, AMPK and Na^+^/K^+^-ATPase in mammals [5], NF-κB in mammals and *Drosophila* [6,7], the calpain/caspase-7/RhoA pathway in mammals [8], Ca^2+^ signalling in mammals [9,10], Gr21a/Gr63a in insects [11,12], GC-D+ neurons in rodents [13] and SLAC1/protein kinase/ABA-dependent pathways in *Arabidopsis* [14,15]. We have very little knowledge of proteins that unambiguously respond directly to CO_2_. The signalling molecules unambiguously identified to signal directly in response to fluctuating inorganic carbon are the Class III nucleotidylyl cyclases of animals, fungi, and prokaryotes [3], a subset of connexins (where Cx26 is the well-studied archetype) in mammals [16], receptor protein tyrosine phosphatase γ of mammals [17], ubiquitin in mammals [18], PII of *Synechocystis* sp. PCC 6803 [19], PP2C phosphatases of plants and fungi [20], and the MPK4/MPK12/HT1 complex of plants [21].

**Fig. 1:**
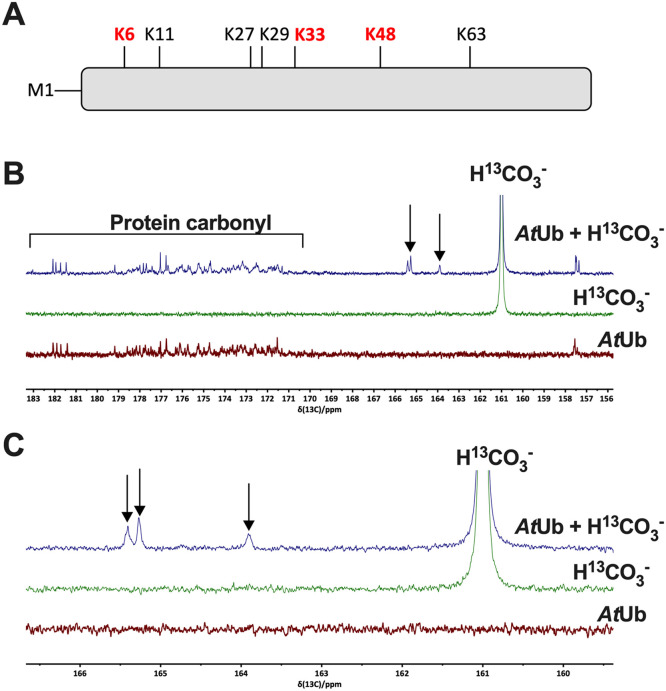
CO_2_ forms carbamates on *At*Ub. **A.** Cartoon of Ub, demonstrating the seven conserved lysine ubiquitination sites and the *N*-terminal M1 site. Those identified as carbamylated through MS/MS are shown in bold red type. **B.** 1D ^13^C-NMR spectra of 5 mM *At*Ub wild type alone, 50 mM NaH^13^CO_3_ alone, and 5 mM *At*Ub wild type with 50 mM NaH^13^CO_3_ are shown. The background H^13^CO_3_^−^ is observed along with carbamates (arrows) and protein carbonyl resonance. **C.** Close-up of the region encompassing the carbamates.

How might CO_2_ regulate and interact with proteins to mediate its physiological effects? CO_2_ can interact with protein to form the carbamate post-translational modification (PTM) on neutral *N*-terminal *α*-amino- or lysine *ε*-amino groups. Carbamylation (Fig. 1A) was initially discovered as a PTM regulating Rubisco [22] and haemoglobin [22] activities. It has subsequently been observed on mammalian ubiquitin [18], cyanobacterial allophycocyanin A [23] and PII protein [19]. Furthermore, a subset of proteins has a locally stabilised carbamate PTM typically utilised for catalysis. Such proteins include urease, alanine racemase, transcarboxylase 5S, class D β-lactamase and phosphotriesterase [24]. On this basis, we have hypothesised that reversible carbamylation of neutral *N*-terminal *α*-amino groups and/or lysine *ε*-amino groups represents a broadly applicable method by which organisms can sense and adapt to fluctuating CO_2_. We have previously deployed triethyloxonium ion in conjunction with tandem mass spectrometry (TEO-MS/MS) to chemically modify carbamate PTMs and enable their identification [24]. TEO is a water-soluble reagent that traps carbamates by selective alkylation. The t_½_ of TEO is approximately 6 min measured at pH 7.4 in an aqueous solution. Therefore, TEO is amenable as a trapping agent to identify protein carbamates as its properties enable its broader use in the laboratory [25]. We have used TEO to identify new CO_2_-binding proteins. Others have exploited the protection offered to lysine by CO_2_ to electrophile modification to enable carbamate identification by a quantitative MS/MS strategy [19].

Studies in prokaryotes [19,23,26] and plants [27] demonstrate multiple proteins that bind CO_2_ through carbamate formation. Therefore, while the identification of MPK4/12 and HT1 as a CO_2_ sensing module that converges on CBC1 kinase in CO_2_-regulated stomatal movements [21] represents a significant advance, it is likely that overall plant physiology responds to CO_2_ by a variety of mechanisms. Therefore, an extended search for plant CO_2_-binding proteins remains a strategically important goal.

Ubiquitin (Ub) is a highly conserved 8.5 kDa protein found in all eukaryotic cells that is conjugated to target proteins resulting in altered activity or targeted degradation in the proteasome. Several enzymes catalyse Ub conjugation. An enzyme cascade mediates Ub conjugation. First, the Ub-activating enzyme (Uba or E1) transfers Ub to the Ub-conjugating enzyme (Ubc or E2) active site, forming a thioester intermediate Ub-E2 [28]. Subsequently, the Ub is transferred from E2, in conjunction with Ub ligase (E3), to specific Lys residues on the surface of a target protein. At least one E1 enzyme, 37 E2 enzymes, and 1,400–1,500 E3 enzymes are predicted to be encoded by the *Arabidopsis* genome [29].

The Ub monomer bound to a protein surface Lys can be potentially conjugated into poly-Ub chains. Such poly-Ub chains have the potential to form on each of the seven Ub lysine side chains and the *N*-terminal α-amino-group, thus permitting many well-defined chain linkages to form [30]. These varying linkages underpin various physiological processes. Proteins bearing K48-conjugated Ub chains are transported to the 26S proteasome for degradation, which regulates numerous plant signalling pathways, including self-incompatibility [31], auxin [32] and jasmonate [33] signalling, and plant immunity [34]. The K63-conjugated K63 functions in apical dominance [35], the DNA damage response [25], and plant immunity [36]. There is little information on poly-Ub chains conjugated on other (M1, K6, K11, K27, K29, K33) sites in plants. The K29-linkage mediates the degradation of DELLA protein degradation [37], and the K11-linkage functions in plant growth and immunity [38].

We have previously identified mammalian Ub as a CO_2_-binding protein and demonstrated that it could explain how CO_2_ regulates the NF-κB pathway [18]. Given the conservation in Ub biochemistry between mammals and plants, we investigated whether CO_2_ regulated plant Ub conjugation. In contrast, to mammalian Ub, whose conjugation is inhibited by [CO_2_] in the mM range, we find that near atmospheric CO_2_ partial pressures enhance plant Ub conjugation.

## 2 Materials and Methods

### 2.1 Ubiquitin expression and purification

The *Arabidopsis thaliana* WT Ub monomer open reading frame was cloned into the NdeI and BamHI sites of pET-23a(+) with a stop codon introduced to preclude the expression of the His_6_ affinity tag. Single lysine to arginine mutant constructs were produced via a commercial gene synthesis/mutagenesis service (Genscript). All *At*Ub variants were expressed as untagged proteins from pET23a in *E. coli* BL21-AI^TM^ (Invitrogen^TM^) at 37°C for 4 h with 400 µM IPTG and 0.1% (w/v) L-arabinose. Pelleted bacteria were resuspended in lysis buffer (50mM Tris-HCl pH 7.6, 1 mM phenylmethylsulfonyl fluoride (PMSF), 0.02% (v/v) NP-40 and 0.4 mg mL^−1^ lysozyme) before being lysed by sonication (180 s on ice) and centrifuged (40,000 x g, 30 min, 4°C). The clarified lysate was transferred to a chilled beaker on ice and stirred vigorously as perchloric acid (PCA) (70% (w/v), 0.35 mL) was added dropwise. The resulting milky solution was left stirring on ice for 10 mins before being centrifuged (20,000 x g, 20 mins, 4°C). The supernatant was dialysed at 4°C for 16 h against 50 mM ammonium acetate pH 4.5 (2 L) with two buffer changes. Dialysed protein was further purified using strong cation exchange chromatography. Using an AKTA Start chromatography system (GE Healthcare), a HiTrap SP HP column (5 mL, Cytiva) was equilibrated with 50 mM ammonium acetate, pH 4.5. The partially purified protein was loaded onto the column and washed with 5 CV equilibration buffer. Protein was then eluted with a NaCl gradient elution (0-500 mM NaCl in 50 mM ammonium acetate buffer, pH 4.5). Fractions were analysed by SDS-PAGE, and the *At*Ub*-*containing fractions were dialysed into storage buffer (20 mM Tris-HCl pH 7.6, 10% glycerol) before being concentrated and snap frozen in liquid N_2_. *At*Ub aliquots were stored at -80°C.

### 2.2 *At*UBA1 expression and purification

The *Arabidopsis thaliana* UBA1 open reading frame was cloned into the NdeI and XhoI sites of pET23a(+) in frame with the *C*-terminal His_6_ tag by commercial gene synthesis (Genscript). *At*UBA1 was expressed as a His_6_-tagged protein from pET23a in *E. coli* BL21-AI^TM^ (Invitrogen^TM^) at 18°C for 20 h with 800 µM IPTG and 0.2% (w/v) L-arabinose. *At*UBA1 was purified by Immobilised Metal Affinity Chromatography (IMAC). The protein was purified using a HisTrap HP column (1 mL, Cytiva) and an equilibration buffer of 50 mM Tris-HCl pH 7.5, 400 mM NaCl, and 20 mM imidazole. The wash and elution buffers consisted of 50 mM Tris-HCl, 400 mM NaCl and 40 mM/200 mM imidazole. *At*UBA1-containing fractions were dialysed into anion exchange start buffer (50 mM Tris-HCl, 50 mM NaCl, 1 mM DTT, 5% (v/v) glycerol) and purified using a HiTrap Q HP column (1 mL, Cytiva). The protein was eluted using a NaCl gradient (50-500 mM), and the resulting fractions were analysed by SDS-PAGE. *At*UBA1 containing fractions were dialysed into size exclusion chromatography buffer (50 mM Tris-HCl, 200 mM NaCl, 1 mM DTT, 15% (v/v) glycerol), concentrated and then further purified by size exclusion chromatography on a 16/600 Superdex 200 pg column (GE Lifesciences) and an AKTA Pure chromatography system.

### 2.3 *At*UBC5 expression and purification

*At*UBC5 was expressed as a His_6_-tagged protein in *E. coli* Rosetta^TM^ 2 (DE3) (Novagen) at 20°C for 20 h with 200 µM IPTG. *At*UBC5 was purified by IMAC. The protein was purified using a HisTrap HP column (1 mL, Cytiva) and an equilibration buffer of 50 mM Tris-HCl pH 7.5, 400 mM NaCl, and 20 mM imidazole. The wash and elution buffers consisted of 50 mM Tris-HCl, 400 mM NaCl and 40 mM/200 mM imidazole. *AtUBC5-containing* fractions were dialysed into anion exchange start buffer (50 mM Tris-HCl, 50 mM NaCl, 1 mM DTT, 5% (v/v) glycerol) and purified using a HiTrap Q HP column (1 mL, Cytiva). The protein was eluted using a NaCl gradient elution (50-500 mM), and the resulting fractions were analysed by SDS-PAGE. *AtUBC5-containing* fractions were dialysed into storage buffer (50 mM Tris-HCl, 200 mM NaCl, 1 mM DTT, 15% (v/v) glycerol), concentrated and stored at -80°C.

### 2.4 Protein CO_2_ trapping

The recombinant protein (50-500 µg) was diluted into 2.5 mL trapping buffer (100 mM NaH_2_PO_4_/Na_2_HPO_4_, 100 mM NaCl, pH 7.4) for CO_2_ trapping experiments. NaHCO_3_ dissolved in trapping buffer (0.5 mL) was added to a final concentration specified for each reaction. The required NaHCO_3_ concentration was determined based on the desired CO_2_ concentration and the solution pH using the Henderson-Hasselbalch equation. This solution was added to a potentiometric titrator (902 Titrando; Metrohm) and incubated at 25°C with stirring for 5 min. A freshly made solution of triethyloxonium (TEO) tetrafluoroborate (240 mg, 1.47 mmol) was prepared in trapping buffer (1 mL) and added dropwise to the solution with constant stirring. The pH was maintained at the desired set point via the automated addition of NaOH (1 M), and the reaction was left stirring for 1 h to ensure complete hydrolysis of the TEO. The trapped solution was then dialysed into dH_2_O (4°C, 16 h) with two buffer changes to ensure the removal of all hydrolysed TEO. The dialysed sample was then dried at room temperature using a centrifugal vacuum concentrator.

### 2.5 Mass spectrometry

S-Trap™ (Protifi) Mini digestion was performed according to the manufacturer's instructions with slight modifications. All steps used LC-MS grade reagents. Dried protein (∼100-300 µg) was resuspended in 1x SDS Lysis buffer (5 % (v/v) SDS, 50 mM triethylammonium bicarbonate (TEAB) pH 8.5, 50 µL). Liquid samples were diluted in an equal volume 2x SDS Lysis Buffer (10% (w/v) SDS, 100 mM TEAB pH 8.5). DTT (20 mM) was added, and the sample was boiled (10 min, 95°C) to reduce disulfide bonds. Iodoacetamide (40 mM) was added, and the sample was incubated in the dark (30 min) to alkylate the sample fully. Phosphoric acid (∼1.2%) was added to the supernatant before the addition of S-Trap Binding Buffer (90 % (v/v) methanol, 100 mM TEAB pH 7.55, 6x total sample volume). The resulting colloidal solution was loaded onto the S-Trap Mini spin column and centrifuged (4000 x g, 30 s) to bind the protein to the S-trap. The column was then washed with S-Trap binding buffer (400 µL) and centrifuged (4000 x g, 30 s). This washing was carried out five times before the S-Trap Mini spin column was transferred to a clean collection tube. A freshly made digestion solution prepared of Trypsin Gold (Promega, Mass Spectrometry Grade) (1:20 (w/w) Trypsin Gold: Sample) in digestion buffer (50 mM TEAB, pH 8.5, 125 µL total volume), was added to the column. The column was briefly centrifuged (4000 x g, 2 s), any flow-through reloaded onto the column and then incubated (37°C, 16 h). Peptides were eluted from the column by adding three elution buffers, with each addition followed by a centrifugation step (1000 x g, 60 s). Elution Buffer 1 (50 mM TEAB, pH 8.5, 80 µL) was used to elute most of the aqueous peptides, followed by Elution Buffer 2 (0.2% (v/v) Formic acid, 80 µL). Finally, Elution Buffer 3 (50% (v/v) acetonitrile (ACN), 0.2% (v/v) formic acid, 80 µL) was used to elute hydrophobic peptides. All eluted peptides were combined and dried at room temperature using a vacuum centrifuge.

The digested peptides were desalted on a C18 column and analysed by ESI-MS/MS on an LTQ Orbitrap XL mass spectrometer (Thermo) coupled to an Ultimate 3000 nano-HPLC instrument. Peptides eluted from the LC gradient were injected online to the mass spectrometer (lock mass enabled, mass range 400–1800 Da, resolution 60,000 at 400 Da, 10 MS/MS spectra per cycle, collision-induced dissociation (CID) at 35% normalised CE, rejection of singly charged ions).

The LC-MS/MS raw data files (.wiff) were converted into .mgf or .mzXML files using the freeware MSConvert (ProteoWizard) and analysed using PEAKS Studio 10.5 software. An error tolerance of 15.0 ppm for the precursor mass using the monoisotopic mass and 0.2 Da for the fragment ion was used. Tryptic digests were selected using a semispecific digest mode and a maximum of three missed cleavages per peptide. Protein modifications used were fixed (57.0215 Da@C) or variable (15.9949 Da@M, 42.0106 Da@*N*-term/K, 72.0211 Da@*N-*term/K, 73.0211 Da@*N-*term/K, 28.0313 Da@*N-*term/D/E/K, 114.0429 Da@T/S/C/K, and 383.23 Da@K).

### 2.6 ^13^C-Nuclear magnetic resonance

Protein was exchanged into NMR sample buffer (100 mM NaH_2_PO_4_/Na_2_HPO_4_, pH 7.6, 100 mM NaCl) using a centrifugal concentrator (Vivaspin Sartorius). The total sample volume was 0.7 mL with protein. Samples contained 10% (v/v) D_2_O, and inorganic carbon was added as NaHCO_3_. ^13^C-NMR spectra were acquired with a Varian 600 MHz spectrometer equipped with an Agilent OneNMR Probe to deliver a maximum pulsed-field gradient strength of 62 G cm^−1^. A ^1^H spectrum was acquired to examine for small molecule impurities. Thirteen ^1^H experiments were recorded in 12 h, collecting 131072 complex points. The repetition time was 6.7 s, of which 1.7 s comprised the acquisition time. The excitation pulse angle was set to 45 degrees. The strong interfering H_2_O signal was eliminated using the Robust-5 pulse sequence. Thirty-two ^13^C scans were collected, comprising 65 536 complex data points and a spectral width of 10 kHz. The repetition time was 6.3 s, of which 3.3 s comprised the acquisition time. The W5 inter-pulse delay was set to 240 µs. Rectangular 1 ms pulsed-field gradients were used in all cases with a strength of G1 = 28.3 G cm^−1^ (first pair) and G2 = 4.9 G cm^−1^ (second pair). The gradient stabilisation delay was 0.5 ms. The first pair of lock pre-focusing field gradients were separated from the first radio-frequency pulse by a 1.5 ms delay.

### 2.7 *In vitro* ubiquitin conjugation assays

Assay components were degassed via sparging with N_2,_ and assays were carried out in an inert atmosphere of N_2_ within an anaerobic chamber (BelleTechnology). Ub conjugation assays were performed in 50 µL reactions in a reaction buffer consisting of 200 mM HEPES pH 7.9, 50 mM KCl, 5 mM MgCl_2_, 2 mM adenosine 5’-triphosphate (ATP), 1 mM DTT, 25 µM *At*Ub, 24 µM *At*UBC5 and 0.1 µM *At*UBA1. C*_i_* was added as HCO_3_^−^/CO_2_, with [anion] maintained with supplemental Cl^−^. Assays were initiated by adding either *At*UBA1 or ATP. Following initiation, assays were incubated (30 min, 25°C) before termination with 2x Laemlli sample buffer. Time course assays were performed as above but in 100 µL reactions. 10 µL aliquots were removed at each time point and terminated as above. Assays were resolved via SDS-PAGE on 15% (w/v) polyacrylamide gels and, following imaging, were analysed using ImageJ software. For normalisation, independent paired experiments (0 versus 100 μM CO_2_) were processed by SDS-PAGE. For each SDS-PAGE gel, the *At*UBC5-*At*Ub conjugate and (*At*UBC5-*At*Ub + free *At*Ub) were quantified. The ratio obtained for 100 μM CO_2_ was normalised to 0 μM CO_2_ = 1. The datasets presented represent the independent paired experiments. Di-Ub linkage analysis was performed by LC-MS/MS following a gel band digest of the di-Ub band. Data were analysed using PEAKS searching for the DiGly variable modification (+114.04 Da) on lysine residues.

### 2.8 Statistical analysis

All data are presented as the distribution of independent data points representing independent experiments. All statistics and graphical analyses were performed using GraphPad Prism 8 (GraphPad Software, inc.).

## 3 Results

### 3.1 CO_2_ forms carbamates on plant ubiquitin

Carbamate formation on mammalian Ub at physiologically relevant CO_2_ partial pressure (PCO_2_) down-regulates Ub cross-linking [18]. A key feature of Ub is its seven conserved lysine residues (K6, K11, K27, K29, K33, K48, K63) and an *N*-terminal α-amino group, which can serve as ubiquitination sites in the formation of poly-Ub chains. These sites are conserved between mammals and plants (Fig. 1A). Therefore, we investigated whether this phenomenon was conserved in a plant Ub of *Arabidopsis thaliana* typically exposed to lower PCO_2_. The open reading frame of a single Ub monomer from *Arabidopsis* was cloned into the expression vector pET23a and purified as a recombinant protein (*At*Ub) (**Supplementary Fig. 1**).

^13^C NMR is a spectroscopic technique used to analyse the environment of carbon atoms within a molecule. 1D ^13^C NMR has been used to report the formation of the carbamate groups on proteins [39,40]. Protein carbonyl resonances are found in the 170-185 ppm region, whereas carbamate resonances are located upfield of these values (163-166 ppm) as the carbon atom experiences relatively high shielding due to the delocalised nature of the negative charge between the heteroatoms of the carbamate group.

^13^C NMR spectra supported the formation of carbamates on *At*Ub (Fig. 1B-C). Within the range of chemical shifts observed, the total inorganic carbon (CO_2_ + HCO_3_ = C*i*) control (green) spectrum consists of a strong HCO_3_^−^ resonance at 161 ppm, while the Ub control spectrum (red) shows the protein carbonyl resonances between 171-182 ppm that are observed in the absence of additional C*_i_*. No resonances are observed between 163-166 ppm, the region associated with carbamate groups, in these spectra. The *At*Ub + C*_i_* spectrum (blue) contains three additional peaks within this range, at 163.9, 165.2 and 165.4 ppm chemical shifts, respectively. The absence of these peaks in the control spectra is evidence that they are derived from interactions between *At*Ub and CO_2_. Furthermore, the spectra were normalised based on the intensity of the protein carbonyl peaks (171-182 ppm). Therefore, the carbamate resonances have not arisen from a difference in the concentration of the Ub samples. The presence of three distinct resonances suggests the formation of more than one distinct carbamate group. There are eight predicted potential sites of carbamate formation on Ub: the ε-amino groups of the seven lysine residues and the α-amino group of the *N*-terminus. However, this spectrum alone cannot assign the carbamate resonances to any of these groups.

### 3.2 Plant ubiquitin binds near atmospheric CO_2_

Following the observation of carbamate groups in their native form on *At*Ub by ^13^C NMR, an orthologous approach was used to identify the specific sites of carbamate formation. Carbamate trapping uses TEO, a Meerwein reagent, to ethylate carbamate groups so they are stable for electrospray ionisation and LC-MS/MS analysis [24]. The carboxyethyl group is identified as a 72.0211 Da mass shift associated with a given lysine residue when analysed by MS/MS.

**Fig. 2:**
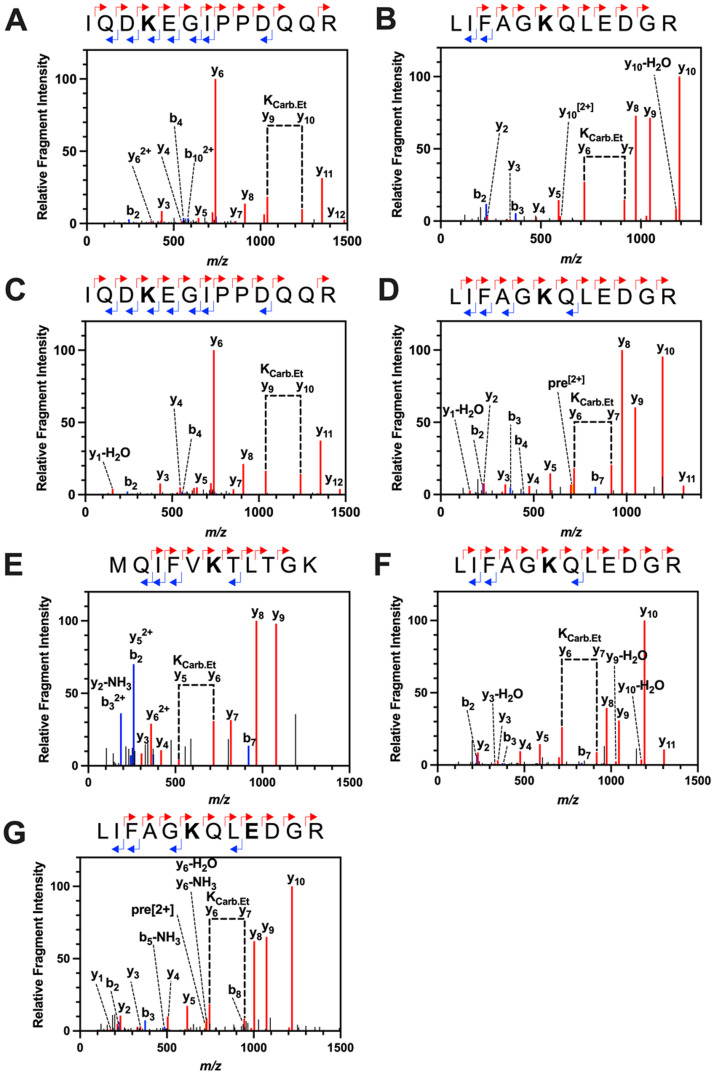
CO_2_ binds *At*Ub. Plots of relative fragment intensity versus mass/charge ratio (m/z) for fragmentation data from MS/MS identifying ethyl-trapped carbamate on recombinant *At*Ub. **A.***At*Ub K33 with 20 mM ^12^CO_2_/HCO_3_^−^. **B.***At*Ub K48 with 20 mM ^12^CO_2_/HCO_3_^−^. **C.***At*Ub K33 with 20 mM ^13^CO_2_/HCO_3_^−^. **D.***At*Ub K48 with 20 mM ^13^CO_2_/HCO_3_^−^. **E.***At*Ub K6 with 130 μM ^12^CO_2_/HCO_3_^−^. **F.***At*Ub K48 with 130 μM ^12^CO_2_/HCO_3_^−^. **G.***At*Ub K48 with 130 μM ^13^CO_2_/HCO_3_^−^. Peptide sequences indicate predominant +1y (red) +1b (blue) ions identified by MS/MS shown in the plot. The modified residue is indicated in bold. K_carb.Et_ indicates the molecular weight difference between ions diagnostic of the modified Lys.

A TEO-MS/MS trapping experiment was carried out on recombinant *At*Ub at elevated C*_i_* (1.47 mM CO_2_). These conditions were have previously been used to screen the *Arabidopsis* proteome for CO_2_-binding sites. Trypsin was used to digest the trapping reaction mixture, and LC-MS/MS was used to analyse samples, followed by data analysis using Peaks (Bioinformatics Solutions Inc). The data were interrogated for modifications on the *N*-terminus and lysine with masses of 72.0211 Da (trapped carbamate) and 28.0313 Da (*O*-ethylation on glutamate and aspartate side chains). The carbamate modification was observed on two *At*Ub lysine residues: K33 and K48 (MS-MS peptide amino acids 30-42 IQDKEGIPPDQQR, proposed carbamylation on K33; MS-MS peptide amino acids 43-54 LIFAGKQLEDGR, proposed carbamylation on K48) (Fig. 2A-B). Within the data sets presented here, the carbamates were observed on both peptides on internal lysine residues, exhibiting a so-called missed cleavage. The missed cleavage occurs because carboxyethylation removes the cationic charge on the lysine essential for cleavage site recognition by trypsin. This observation supports the identification of carbamates on both *At*Ub K33 and K48 as a missed cleavage is an otherwise rare event. Peptides covering four of the remaining lysines (all but K6) were identified, suggesting carbamates do not form on these lysines at 1.47 mM C*i*. However, we cannot eliminate the possibility that carboxyethylated peptides at these sites are refractory to MS/MS.

As an additional control, the experiment was repeated with the same concentration of ^13^C*_i_*, in which the carboxyethylation modification shows a 73.0211 Da m/z shift due to the additional +1 Da m/z of the ^13^C isotope. Peptides containing a ^13^C-carboxyethylated lysine were observed for both the K33 and K48 residues, validating both sites (Fig. 2C-D).

Despite observing the carbamate modification on K33 and K48 residues at 1.47 mM CO_2_, this is orders of magnitude higher than physiologically relevant PCO_2_ for *Arabidopsis*. Cellular [CO_2_] within typical C3 plants is much closer to atmospheric CO_2_ (∼10 µM) [41], and thus *At*Ub was trapped under these conditions. Independent experiments identified the carboxyethyl modification on the K6 and K48 residues of *At*Ub (Fig. 2E-F). The carboxyethyl modification was not observed on the K33 residue under these conditions, while unmodified K33-containing peptides were identified. This finding suggests that the K33 residue is less sensitive to CO_2_ than the K6 and K48 residues and that the K33 carbamate group only forms at elevated PCO_2_.

A subsequent trapping experiment with ^13^C*_i_* identified the ^13^C carboxyethyl modification on K48 (Fig. 2G). The K6 residue was not identified with near atmospheric ^13^CO_2_. However, we note that the *At*Ub *N-*terminal peptide was typically unreliable to observe by MS/MS, suggesting that the modified and unmodified forms of this peptide exhibit relatively poor flyability [42].

### 3.3 Near-atmospheric CO_2_ stimulates plant Ub conjugation

Having identified carbamylation sites at K6 and K48 of *At*Ub sensitive to micromolar CO_2_, we developed an *in vitro* assay system to test the potential biochemical impact of these modifications. There are two Ub-activating E1 enzymes in *A. thaliana: At*UBA1 and *At*UBA2, which are reported to display broadly similar expression profiles across the plant [43]. *At*UBA1 has previously been recombinantly expressed and widely used in plant *in vitro* ubiquitination assays and thus was selected as the E1 enzyme for this work. The *A. thaliana* E2 enzyme *At*UBC5 is one of nine reported to function in an E3-dependent manner [44]. *At*UBA1 and *At*UBC5 were produced as purified recombinant proteins (**Supplementary Fig. 2**), and we confirmed the formation of di-*At*Ub was dependent on *At*UBA1, *At*UBC5, *At*Ub, and ATP (**Supplementary Fig. 3**).

**Fig. 3:**
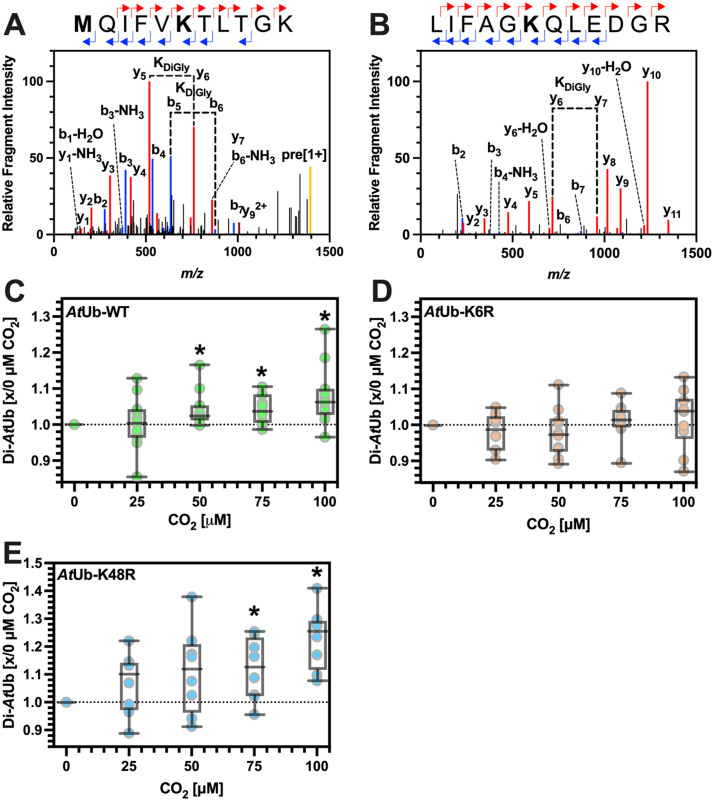
CO_2_ increases *At*Ub conjugation. **A-B.** Plots of relative fragment intensity versus mass/charge ratio (m/z) for fragmentation data from MS/MS identifying the di-Gly modification on recombinant *At*Ub at K6 (**A**) and K48 (**B**). Peptide sequences indicate predominant +1y (red) +1b (blue) ions identified by MS/MS shown in the plot. The modified residue is indicated in bold. K_Digly_ indicates the molecular weight difference between ions diagnostic of the modified Lys. **C-E.** Box and whisker (5-95%) plots of the ratio of di-*At*Ub formed in the presence of added CO_2_ compared to zero CO_2_. **C.***At*Ub-WT conjugation (* p<0.05, one-sample t-test, theoretical mean=1.000; 25 μM CO_2_*p* = 0.8531, t=0.1900, df=10; 50 μM CO_2_*p* = 0.0163, t=2.884, df=10; 75 μM CO_2_*p* = 0.0059, t=3.486, df=10; 100 μM CO_2_*p* = 0.0121, t=3.056, df=10). **D.***At*Ub-K6R conjugation (one-sample t-test, theoretical mean=1.000; 25 μM CO_2_*p* = 0.3027, t=1.093, df=9; 50 μM CO_2_*p* = 0.3440, t=0.9988, df=9; 75 μM CO_2_*p* = 0.4587, t=0.7742, df=9; 100 μM CO_2_*p* = 0.5219, t=0.6664, df=9). **E.***At*Ub-K48R conjugation (* p<0.05, one-sample t-test, theoretical mean=1.000; 25 μM CO_2_*p* = 0.1246, t=1.744, df=7; 50 μM CO_2_*p* = 0.0904, t=2.043, df=7; 75 μM CO_2_*p* = 0.0199, t=3.002, df=7; 100 μM CO_2_*p* = 0.0006, t=5.900, df=7).

We determined the di-*At*Ub linkage type in assays by LC-MS/MS and searched for a diglycine +114.04 Da mass shift formed after trypsin cleavage at the *At*Ub *C-*terminus (**Supplementary Fig. 4**). LC-MS/MS analysis of trypsin-digested *At*UBC5-synthesised di-*At*Ub identified the +114.04 Da di-glycine modification on K6 and K48 (Fig. 3A-B). Unmodified peptides containing all the other *At*Ub lysine residues were identified, indicating *At*UBC5 does not conjugate *At*Ub at these residues. Repeated experiments demonstrated that the +114.04 Da di-glycine modification on K6 was not observed using *At*Ub-K6R protein. However, the +114.04 Da di-glycine modification on K48 was still observed using *At*Ub-K6R protein. The +114.04 Da di-glycine modification on K48 was not observed using *At*Ub-K48R protein. The +114.04 Da di-glycine modification on K6 was still observed using *At*Ub-K48R protein. Therefore, K6R and K48R *At*Ub mutant proteins were used to analyse the relative contributions of these two linkage types to di-*At*Ub formation. Di-*At*Ub production was measured as a proportion of the sum of mono- and di-*At*Ub. For each mutant, this was then normalised to di-*At*Ub production with the WT protein. For *At*Ub-K6R, the mean normalised intensity of the di-*At*Ub band was 88.1% of the WT di-*At*Ub band. For *At*Ub-K48R, the mean normalised intensity of the di-*At*Ub band was 4.4% of the WT di-*At*Ub band. We conclude that under the standard conditions of the assay, *At*UBC5 exhibits a strong preference for forming K48-conjugated di-*At*Ub *in vitro*. The enzyme further produces a relatively small population of K6-conjugated di-*At*Ub. Therefore, *At*UBC5 is suitable for investigating the impact of carbamate formation at K6 and K48 at near atmospheric PCO_2_.

The pH sensitivity of the *At*UBA1/*At*UBC5 system was measured across a range of pH values (pH 6.5-8.3), and we observed a clear pH dependence (**Supplementary Fig. 5**). Degassing CO_2_ from an assay can increase pH and give the appearance of a stimulated activity. Therefore, we performed assays to assess the role of CO_2_ at pH 7.9, close to the observed pH optimum. Further, we monitored assay pH over a PCO_2_ range (1-4 mM total C*_i_*) and demonstrated no difference in final assay pH (7.8). We could, therefore, eliminate altered pH as an explanation for any observations. To probe the effects of *At*Ub carbamylation on di-*At*Ub formation, *At*UBC5-catalysed di-*At*Ub formation was measured at increasing PCO_2_ (Fig. 3C). We observed a statistically significant increase in the mean rate of di-*At*Ub formation with increasing PCO_2_. This surprising result was in direct contrast to previous observations, where significantly higher PCO_2_ inhibited mammalian Ub conjugation.

Having observed this unexpected stimulation in di-*At*Ub formation using WT *At*Ub, K6R and K48R mutant *At*Ub were used to determine whether carbamylation at either residue was responsible for this effect (Fig. 3D-E). *At*Ub-K6R ablated the stimulation in di-*At*Ub formation across the same PCO_2_ range. There was no statistically significant difference in the mean of the normalised values at each PCO_2_ relative to the zero PCO_2_ values. This observation suggests that the CO_2_-stimulated increase in di-*At*Ub formation depends on K6. Conversely, the CO_2_-stimulated increase in di-*At*Ub formation was still observed using *At*Ub-K48R, presumably through an increase in conjugation at the K6 site.

To control for CO_2_-mediated effects via carbamylation of *At*UBA1 and *At*UBC5, both enzymes were TEO-trapped at 100 µM CO_2_, the highest [CO_2_] used within the *in vitro* assays. No carboxyethyl modifications were observed on *At*UBC5 under these conditions (89 peptides contributing to 83% protein coverage at a 1% false discovery rate). The only lysine outside of the covered region was the *At*UBC5 K5 residue. The absence of carboxyethyl modifications suggests that any observed effects are not due to carbamate formation on *At*UBC5. However, it is not possible to formally exclude *At*UBC5 K5 carbamylation. Similarly, no carboxyethyl modifications were observed on *At*UBA1 under these conditions (299 peptides contributing to 91% protein coverage at a 1% false discovery rate). Unfortunately, multiple lysine residues were not covered by the identified peptides due to the proximity of these lysine residues to other lysine and arginine residues. Where lysine and arginine residues are in close proximity in a protein, very small peptides result after trypsin digest. Such small peptides are very difficult to identify by MS/MS.

### 3.4 Near-atmospheric CO_2_ stimulates plant UBC5 charging

Incomplete MS/MS coverage of *At*UBA1 and *At*UBC5 cannot formally exclude a CO_2_ effect on either or both enzymes. However, the observation that *At*Ub K6R ablates the CO_2_ response makes such a possibility unlikely. Therefore, we investigated whether the *At*UBA1 and *At*UBC5 charging steps explained the observations dependent on *At*Ub carbamylation. The charging reaction was investigated in which *At*UBA1 adenylates the *At*Ub *C*-terminus, activating it for transfer to a cysteine at the *At*UBA1 active site via a thioesterification reaction. Even at 4°C, by the first assay time point (at 5s), the concentration of the *At*UBA1-*At*Ub conjugate had reached a steady state (**Supplementary Fig. 6**). Thus, the rate of this step relative to the others within the reaction made it unlikely it could be responsible for a pronounced change in di-*At*Ub conjugation in response to CO_2_.

The *At*UBC5 charging step occurs via a transthioesterification reaction in which the *At*Ub is transferred from the E1 active site cysteine to the E2 active site cysteine via forming an E1-E2-Ub complex. The exact mechanism remains uncharacterised, but recent structural studies using a stable Cdc34-Ub mimetic in complex with UBA1 have shed light on two distinct conformations the complex can occupy, potentially providing two snapshots of the transthioesterification mechanism [45]. In di-*At*Ub formation, the *At*Ub-*At*UBC5 conjugate can be considered the active form E2 enzyme. Therefore, we investigated whether *At*Ub carbamylation influences *At*Ub-*At*UBC5 conjugate formation.

**Fig. 4:**
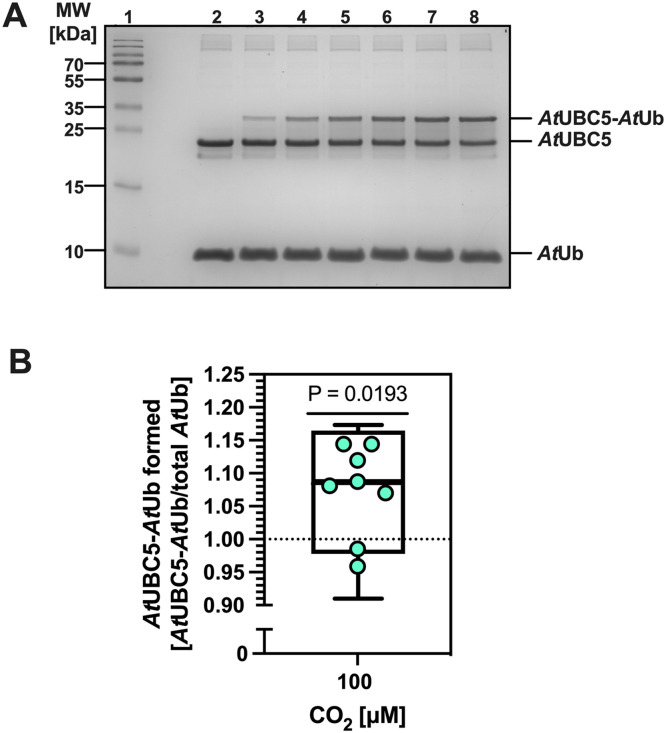
CO_2_ increases *At*UBC5 charging. **A.** Example of SDS/PAGE analysis and Coomassie Blue staining showing the time course of *At*UBC5-*At*Ub conjugate formation performed at atmospheric CO_2_. Lanes are 1. Molecular mass standards; 2. Time zero; 3. 1 min; 4. 2 mins; 5. 3 mins; 6. 4 mins; 7. 5 mins; 8. 6 mins. **B.** Box and whisker (5-95%) plot of *At*UBC5-*At*Ub conjugate formed at 100 μM CO_2_ when *At*UBC5-*At*Ub conjugate formation at zero CO_2_ is normalized to 1 (*p* = 0.0193, one-sample t-test, theoretical mean=1.000; 100 μM CO_2_, t=3.025, df=7). Assays were run for two minutes.

The rate of *At*UBC5-*At*Ub conjugation was measured at 0 and 100 µM CO_2_ (the upper and lower bounds of the concentrations used in the di-*At*Ub conjugation reaction (Fig. 3)). We observed a statistically significant increase (∼9%) in the mean ratio of *At*UBC5-*At*Ub conjugate formation at 100 µM CO_2_ relative to 0 µM (Fig. 4). Therefore, *At*Ub carbamylation at K6 increases di-*At*Ub formation by enhancing *At*UBC5-*At*Ub formation.

## 4 Discussion

TEO-MS/MS with low micromolar CO_2_ concentrations identified the *At*Ub K48 and K6 residues as CO_2_-binding targets. Observing these sites under physiologically relevant CO_2_ partial pressures suggests they may be biochemically relevant post-translational modifications within a plant. The identification of the carbamate PTM on the K6 residue is supported by analysis of Ub lysine side chain p*K*_a_ values via HSQC experiments that predicted the K6 residue to have a moderately suppressed p*K*_aH_ relative to other Ub lysine residues [46] (where p*K*_aH_ represents the p*K*_a_ of the conjugate acid used to determine amine basicity). The Ub K48 residue had the second lowest predicted p*K*_aH._ These two sites were identified at atmospheric CO_2,_ indicating a correlation between side chain p*K*_aH_ and sensitivity to carbamylation, as previously postulated [22]. However, while these p*K*_aH_ values are suppressed relative to the other lysine residues, they are not strikingly low (∼10.5). This value is close to the reported p*K*_aH_ of a free L-lysine side chain. It would still equate to a minor fraction of the ε-amino group existing in the deprotonated state at a physiological pH. Inspection of Ub crystal structures shows that both the K6 and K48 residues are broadly solvent exposed [47,48]. While this solvent exposure would favour interactions with CO_2_, subsequent carbamate stabilising interactions, such as those observed in haemoglobin, appear less plausible. Early studies on Ub acetylation using *p*-nitrophenyl acetate reported complete acetylation of the K6 residue [49]. While carbamylation and acetylation are two distinct processes, they are both non-enzymatic and are underpinned by the nucleophilicity of the ε-amino group. Given the measured p*K*_aH_, it seems there are additional underpinning factors that determine modification.

The E2 enzyme, *At*UBC5, was observed to form free Ub chains conjugated primarily via K48 and K6. Demonstrating *At*UBC5 as a K48-specific plant E2 is intriguing as there is little literature on fully characterised plant E2 enzymes. K48-specific E2 enzymes such as the mammalian E2-25k and the yeast Cdc34 have attracted particular attention due to the canonical role of K48-linked Ub in the 26S-proteasomal degradation pathway [50]. *At*UBC5 could provide a further case study across kingdoms, helping to unpack the mechanisms which determine the linkage specificity by which E2 enzymes synthesise polyubiquitin chains.

*In vitro* assays using the *At*UBC5 indicated that di-*At*Ub formation was stimulated by increasing PCO_2_ in a K6-dependent manner. Limitations to the *in vitro* system meant that while assay CO_2_ concentrations were near atmospheric, they still slightly exceeded those that might be observed within a plant. The molar ratio of *At*Ub to CO_2_ resembled that of theoretical conditions based on mammalian tissue-free Ub concentrations, as the cellular concentration of free Ub in plant tissues is unreported. Therefore, it is hard to perfectly ascribe the *in vitro* observations in this study to a plant cellular environment. However, all reasonable steps have been taken to mimic likely conditions. Nonetheless, plant dark respiration can see rapid rises in leaf intercellular [CO_2_]. Therefore, it is possible that the response to CO_2_ underpins a diurnal regulation of Ub conjugation.

The work of Linthwaite et al. demonstrated that mammalian Ub K48 carbamylation inhibited E2-25k catalysed K48 poly-Ub chain synthesis *in vitro* [18]. This observation was hypothesised to occur due to the carbamylation PTM directly blocking access to the K48 residue of acceptor Ub. That carbamylation could have the opposite effect in the plant K48-specific system appears contradictory but can be explained by distinctions between the assays. Firstly, the finding that inhibition of K48-linked poly-*At*Ub synthesis was not observed during this work can be explained by the significantly lower CO_2_ concentrations used, which do not resemble the concentration range across which Linthwaite et al. observed inhibition. Secondly, that Linthwaite et al. did not observe observe an allosteric stimulation via the K6 residue may be due to differences between the mammalian E1-E2 and plant E1-E2 combinations used in the studies. Consistent with this, Linthwaite et al. identified a Ub carbamate site at K63 by ^13^C-NMR but did not observe inhibition of polyubiquitin chain synthesis with a K63-specific system across the same range of CO_2_ concentrations.

As poly-Ub chain synthesis is a complex multi-step reaction, individual steps were probed to establish how a K6-mediated stimulation in activity might occur. The initial *At*UBA1-*At*Ub (E1-*At*Ub) charging reaction was rapid and unlikely to have a rate-determining effect on di-*At*Ub formation. *k_cat_* values for E1-catalyzed reaction steps have previously been reported in the 1-2 s^−1^ range, over 10-fold greater than reported *k_cat_* values for E2-catalysed ubiquitin conjugation and chain extension reactions [51]. The subsequent Ub transthioesterification step appeared a better candidate. *At*UBC5-*At*Ub thioester conjugate formation was enhanced by increasing CO_2_. How might this occur? Structural studies of mimetic *S. cerevisiae* UBA1-Cdc34-Ub complexes adopted during the transthioesterification reaction displayed hydrogen-bonding interactions between a modified K6R ubiquitin residue and both the UBA1 and Cdc34 enzymes in respective open and closed conformations [45]. While the specific mechanistic features of how K6 carbamylation would stimulate the reaction are unknown, perturbations of these hydrogen-bonding interactions by K6 carbamate formation are a plausible starting point. The proximity of the K6 residue to the Ile44 hydrophobic patch, an essential recognition domain which mediates many of the Ub protein-protein interactions [52,53], might reveal how the K6-dependant CO_2_-mediated stimulation can occur at the Ub-Ub conjugation level. The K6 residue is important in donor Ub docking to the K11-specific E2 Ube2S, forming an ionic interaction with a glutamic acid residue on the Ube2S docking surface [54].

Similarly, in Cdc34, the K6 residue of acceptor Ub interacts with the Cdc34 enzyme via hydrogen bonding to orient the K48 residue towards the E2-Ub thioester bond [55]. A K6D Ub mutation decreased the Ub release rate from the Cdc34-Ub complex. However, this observation was attributed to the length of the aspartic acid side chain and the loss of the polar contact with Cdc34 S71 as opposed to the introduction of the anionic charge, as would be observed for carbamylation. Such allosteric effects have been observed for acetylated Ub residues. Acetyl-K6 and acetyl-K48 Ub were present in low concentrations in human 293F cells, and *in vitro,* both inhibited free poly-Ub chain formation (as catalysed by multiple E2 enzymes) [56]. The authors observed broad repression with acetyl-K6, but acetylation differs from carbamylation in that it irreversibly modifies the cationic lysine residue with a neutrally charged group instead of the transient, anionic carbamate group.

K6 linkages are generally considered atypical in plants [57]. Analysis of the *A. thaliana* ubiquitylome indicated they were present at a low abundance [58,59]. The cellular functions of K6-linked poly-Ub chains also remain uncharacterised in plants, with functional studies limited to other eukaryotes where they have been associated with processes such as DNA repair and mitophagy [60]. Future work should be directed towards understanding the extent to which Ub K6 carbamylation impacts E2 charging and the impact on plant physiology.

## Funding

10.13039/501100000268Biotechnology and Biological Sciences Research Council Grant BB/S015132/1 and a Biotechnology and Biological Sciences Research Council CASE studentship awarded with KWS Saat supported this work.

## CRediT authorship contribution statement

**Harry G Gannon:** Methodology, Validation, Formal analysis, Investigation, Writing – review & editing. **Martin J Cann:** Writing – original draft, Visualization, Supervision, Project administration, Funding acquisition.

## Declaration of Competing Interest

The authors declare that they have no known competing financial interests or personal relationships that could have appeared to influence the work reported in this paper.

## Data Availability

Data will be made available on request.

## Appendix Supplementary materials

Image, application 1
