# Supplementary material for: Near atmospheric carbon dioxide activates plant ubiquitin cross-linking
Source: BBA Adv. 2023 Jun 17;4:100096. doi: 10.1016/j.bbadva.2023.100096 (PMC10319984; doi:10.1016/j.bbadva.2023.100096)
Supplement: Supplementary file 1 [file mmc1.docx]

**Supplementary Data**

**Near atmospheric carbon dioxide activates plant ubiquitin cross-linking**

Harry G Gannon^a^ & Martin J Cann^a,b*^

^a^ Department of Biosciences and ^b^ Biophysical Sciences Institute, Durham University, South Road, Durham, DH1 3LE, United Kingdom

* For correspondence

Email addresses

Harry G Gannon (harry.g.gannon@durham.ac.uk)

Martin J Cann ([m.j.cann@durham.ac.uk](mailto:m.j.cann@durham.ac.uk))

**Supplementary Figure 1.** Purification of recombinant *At*Ub (SDS/PAGE analysis and Coomassie Blue staining). **1.** Molecular mass standards. **2.** 10 μg whole bacterial lysate. **3.** Purified *At*Ub.

**Supplementary Figure 2.** Purification of recombinant proteins (SDS/PAGE analysis and Coomassie Blue staining). **1.** Molecular mass standards. **2.** *At*UBA1. **3.** *At*UBC5. **4.** *At*Ub.

**Supplementary Figure 3.** An *in vitro* *At*Ub conjugation assay requiring *At*UBA1, *At*UBC5, *At*Ub, and ATP. **1.** Molecular mass standards. **2.** Assay without *At*UBA1. **3.** All components present. **4.** Assay without *At*UBC5. **5.** Assay without *At*Ub. **6.** Assay without ATP. *At*UBC5 charged with *At*Ub (UBC5-Ub) and conjugated di-*At*Ub (Ub_2_) are shown, and their formation depends on all assay components.

**Supplementary Figure 4.** Trypsin cleavage of di-*At*Ub at the *C*-terminus of Arg74 of the first *At*Ub monomer (Ub1) leaves a 114.04 Da Gly-Gly group on the conjugated lysine of the second *At*Ub monomer (Ub2). MS/MS can identify this Gly-Gly group.

**Supplementary Figure 5. A.** Plot of di-*At*Ub formation as a fraction of total *At*Ub as a function of pH. Error bars represent S.E.M. (*n*=3) and are smaller than the data points shown. **B**. Plot of assay pH as a function of time at the indicated total inorganic carbon. Error bars represent S.E.M. (*n*=3).

**Supplementary Figure 6.** SDS/PAGE analysis and SYPRO™ Ruby (Invitrogen) staining showing the time course of *At*UBA1-*At*Ub conjugate formation. Lanes are 1. Molecular mass standards; 2. Time zero; 3. 5 s; 4. 10 s; 5. 30 s; 6. 60 s; 7. 90 s; 8. 120 s; 9. 180 s; 10. 300 s.
